# β-CATENIN is a positive prognostic marker for HPV-positive head and neck squamous cell carcinoma

**DOI:** 10.1007/s00432-023-04712-3

**Published:** 2023-04-03

**Authors:** Stefan Stoiber, Faris F. Brkic, Tobias Maier, Julia Schnoell, Elisabeth Gurnhofer, Gregor Heiduschka, Lorenz Kadletz-Wanke, Lukas Kenner

**Affiliations:** 1 Department of Pathology, Medical University of Vienna, Vienna, Austria; 2 Christian Doppler Laboratory for Applied Metabolomics, Vienna, Austria; 3 Department of Otorhinolaryngology and Head and Neck Surgery, Medical University of Vienna, Vienna, Austria; 4 Unit of Laboratory Animal Pathology, University of Veterinary Medicine, Vienna, Austria; 5 CBmed GmbH—Center for Biomarker Research in Medicine, Graz, Austria

**Keywords:** β-Catenin, WnT/β-Catenin pathway, HPV, Head and neck cancer, Immunohistochemistry

## Abstract

**Purpose:**

The evolutionary-conserved Wnt/β-CATENIN (WBC) pathway has been implicated in the pathogenesis of different solid malignant tumors. We evaluated the prognostic relevance of β-CATENIN, a pivotal mediator of WBC activation, in patients with human papillomavirus (HPV)-positive head and neck squamous cell carcinoma (HNSCC).

**Methods:**

We analyzed if patients with HPV-positive HNSCC from the “The Cancer Genome Atlas” (TCGA cohort, *n* = *41*) can be stratified based on their *CTNNB1* mRNA expression. Moreover, in a tissue microarray (TMA) of primary tumor sections from HPV-positive HNSCC patients treated in a tertiary academic center (in-house cohort, *n* = 31), we evaluated the prognostic relevance of β-CATENIN expression on protein level.

**Results:**

In silico mining of *CTNNB1* expression in HPV-positive HNSCC revealed that high *CTNNB1* expression was linked to better overall survival (OS, *p* = 0.062). Moreover, high β-CATENIN expression was significantly associated with a better OS in our in-house cohort (*p* = 0.035).

**Conclusion:**

Based on these findings, we postulate that β-CATENIN expression could serve (potentially in conjunction with other WBC pathway members) as a marker for better survival outcomes in patients with HPV-positive HNSCC. However, it is evident that future studies on bigger cohorts are warranted.

**Supplementary Information:**

The online version contains supplementary material available at 10.1007/s00432-023-04712-3.

## Introduction

Head and neck squamous cell carcinoma (HNSCC) originate from the mucosal linings of the upper aerodigestive tract. In 2020, approximately 870,000 new cases were recorded, making HNSCC the seventh most common cancer worldwide (Sung et al. 2021). Habits, such as smoking and alcohol consumption, chronically expose the mucosa of the aerodigestive tract to environmental carcinogens, which are a major contributor to HNSCC tumorigenesis. Although the numbers of smokers and drinkers are globally gradually decreasing (Brkic et al. 2021), an increase of the relative incidence rate of oropharyngeal squamous cell carcinoma (OPSCC) has been observed. This is most likely linked to the rise in human papillomavirus (HPV) infections (Brkic et al. 2021). For the last 2 decades, the role and importance of the DNA oncovirus HPV in OPSCC has been well illustrated, particularly for the high-risk variant “HPV-16” (Elrefaey et al. 2014a; Ward et al. 2014). HPV has epitheliotropic properties, meaning it has an affinity for epithelial tissue (Elrefaey et al. 2014a). Especially the local crypts of the oropharynx facilitate its replication, hence there is an inherent propensity of HPV to infect the epithelium of the oropharynx (Elrefaey et al. 2014b; Elrefaey et al. 2014a). Despite HPV-positive OPSCC being linked to better survival outcomes, their clinical management does not differ from their HPV-negative counterparts. Until now, the most widely utilized systemic therapy is cisplatin which is highly effective but comes with severe side effects including acute kidney failure, alopecia, and neuropathy (Mehanna et al. 2019). Hence, recently options for treatment de-escalation have been discussed due to the comparatively favorable survival rates of HPV-positive OPSCC (Golusinski et al. 2021). Nonetheless, specific findings related to the identification of novel prognostic markers which could guide treatment decisions are scarce. Intriguingly, classical risk factors, such as T/N stage, do not have the same prognostic value in HPV-positive HNSCC as in HPV-negative HNSCC (Elrefaey et al. 2014b). Therefore, discovering novel markers for patient stratification at early stages might facilitate better patient management and identification of de-escalation treatment candidates.

Wnt/β-CATENIN (WBC) signaling is an important, well-conserved signaling pathway throughout evolution and is implicated in various key cellular processes, such as proliferation, migration, and cell differentiation (Lee et al. 2014; Pai et al. 2017). Due to its diverse roles, a deregulation of the WBC pathway can have profound consequences, such as carcinogenesis. This has already been illustrated for colorectal and ovarian cancer (Zhan et al. 2016). Recent evidence suggests that the activation of canonical WBC signaling plays a role in the disease progression of HPV-negative HNSCC by modulating the migratory and invasive potential of tumor cells (Moon et al. 2021). In contrast, the role of deregulated WBC signaling in HPV-positive HNSCC is still largely unknown.

Multiple lines of evidence suggest that the HPV-specific viral oncoproteins E6 and E7 directly or indirectly interact with β-CATENIN, thereby positively modulating its activity. For example, the viral oncoprotein E6 blocks the proteasomal degradation of β-CATENIN—promoting its nuclear translocation and activation of the pathway. This abnormal increase in WBC pathway activity leads to/can lead to the carcinogenesis of cervical cancer (Wang et al. 2020). Furthermore, Rampias et al*.* (2010) unveiled that the function of seven in absentia homologue (Siah-1), which can promote the degradation of β-CATENIN, is repressed by the viral oncoproteins E6 and E7. This repression of Siah-1 by E6 and E7 indicates their direct role in the nuclear accumulation of β-CATENIN in HPV-associated OPSCC. Interestingly, blockade of the WBC pathway by a specific inhibitor of Creb-binding protein (CBP) has been demonstrated to be specifically effective in the HPV-positive HNSCC cell line SCC154 compared to the HPV-negative HNSCC cell line Cal27 (Brkic et al. 2022). Moreover, MSAB, a small inhibitor of β-CATENIN, has been proven to have antineoplastic effects in HNSCC as well—supporting the oncogenic role of the WBC pathway in HNSCC (Maier et al. 2023).

Based on this status quo, we aimed to investigate the association of WBC signaling with survival in HPV-positive HNSCC. In particular, we aimed to assess the prognostic potential of β-CATENIN expression in two independent cohorts in silico, on mRNA and protein level. These results could potentially facilitate the identification of a new and easily obtainable prognostic marker for HPV-positive HNSCC.

## Materials and methods

### The cancer genome atlas (TCGA)—TCGA cohort

For the analysis of the prognostic value of *CTNNB1* expression, data from all eligible HPV-positive HNSCC patients (= positive status for HPV via fluorescence in situ hybridization (FISH) or positive status for p16 staining) was retrieved using the TCGA database (*n* = *41*, https://portal.gdc.cancer.gov/projects/TCGA-HNSC. Accessed on 1st of April 2022). All available clinical, follow-up and gene expression (Illumina HiSeq RNA sequencing) data from patients with a primary treated and HPV-positive HNSCC were retrieved from the GDC Legacy Archive (https://portal.gdc.cancer.gov/legacy-archive/search/f. Accessed on 30th of December 2021) and normalized utilizing the R package “TCGA biolinks” (version 2.19.0) and R (version 4.0.3, R Foundation for Statistical Computing, Vienna, Austria). Furthermore, the expression values of *CTNNB1* for all 41 patients were filtered and used for the survival analysis (see Statistical analysis section for further details).

### Immunohistochemistry (IHC)—the in-house cohort

Patients with a histologically verified HPV-positive HNSCC, primarily surgically treated in the General Hospital of Vienna between 1st of January 2012 and 31st of December 2017, were included in this investigation. The HPV positivity was determined by FISH. The patients which did not match the inclusion criteria (no recurrent disease and no secondary malignancy) were not considered for the analysis. Besides the tissue, data on age, sex, tumor staging, and other clinically relevant data were retrospectively collected.

For the analysis of the association of β-CATENIN expression with overall survival (OS) and disease-free survival (DFS), a tissue microarray (TMA) of formalin-fixed paraffin-embedded (FFPE) biopsy samples of all eligible patients was constructed. The construction of the TMA was performed with the computer-assisted tissue microarray platform (TMA Grand Master, 3D Histech, Budapest, Hungary). Areas in the FFPE biopsy samples containing predominantly tumor cells were chosen as source material for the TMA cores (general layout of the TMA is shown in supplementary Fig. 1). The immunohistochemical staining was performed on 4 µm slices with the Lab Vision UltraVision Kit (Thermo Scientific, TL-060-HL). Colon tissue sections served as a positive control for the staining. The actual staining was performed as previously described (Brkic et al. 2022) with a 1:50 dilution of the β-CATENIN antibody (Santa Cruz, sc-7963). For the quantification of positive β-CATENIN staining QuPath (Version 0.2.3) was used and the percentage (0–100%) of stained cells for each of the patient TMA cores (3 replicates per patient) was analyzed. Next, the mean of all three cores per patient was calculated and used in the subsequent survival analysis (see Statistical analysis section for further details).

### Statistical anaylsis

The log-rank test for the survival analyses (TCGA cohort and in-house cohort) was performed utilizing the R packages “survival” and “survminer” (versions 3.2.13 and 0.4.9, respectively) and a *p value* below 0.05 was considered as statistically significant. The Kaplan–Meier curves were plotted with “ggplot” (version 3.3.3).

For the patient stratification of the TCGA cohort, the optimized threshold value (OTV) of *CTNNB1* expression regarding OS was calculated using the *surv_cutpoint* function from the “survminer” R package with a patient distribution of at least 20% per group and resulted in a value of 15,943. Based on this value, the patients were stratified and the resulting patient distribution was plotted in a Kaplan–Meier curve.

For the patient stratification of the in-house cohort the OTVs, with regards to OS and DFS, were calculated (*surv_cutpoint* function from the “survminer” R package) and used as a cutoffs (> OTV was considered as high). The calculation of the OTV with a patient distribution of at least 20% per group resulted in a cutoff of 7.33%, for OS and DFS, respectively. We plotted the Kaplan-Meier curves with “ggplot”.

## Results

### High expression of CTNNB1 mRNA is associated with a better overall survival—TCGA cohort

*CTNNB1* expression and clinical data of 41 HPV-positive HNSCC patients were retrieved from the TCGA database. The characteristics of the whole cohort, as well as the subgroups—post-stratification based on the OTV of *CTNNB1* expression—are shown in Table 1.

**Table 1:** Patient characteristics of HPV-positive HNSCC patients of the TCGA cohort—stratified by *CTNNB1* expression

Characteristic	*CTNNB1* low	*CTNNB1* high	Total
Number of patients (%)	23	18	41
Age, median (years)	57.4	53.5	57.0
Range (years)	41.1–68.3	40.6–71.5	40.6–71.5
T stage	*n* (%)	*n* (%)	*n* (%)
T4	6 (14.6)	2 (4.9)	8 (19.5)
T3	3 (7.3)	2 (4.9)	5 (12.2)
T2	12 (29.3)	9 (21.9)	21 (51.2)
T1	2 (4.9)	4 (9.8)	6 (14.7)
Tx	0 (0.0)	1 (2.4)	2 (2.4)
N stage	*n* (%)	*n* (%)	*n* (%)
N3	0 (0.0)	1 (2.4)	1 (2.4)
N2	16 (39.0)	10 (24.4)	26 (63.4)
N1	2 (4.9)	3 (7.3)	5 (12.2)
N0	4 (9.8)	4 (9.8)	8 (19.6)
Nx	0 (0.0)	1 (2.4)	1 (2.4)
M stage	*n* (%)	*n* (%)	*n* (%)
M0	21 (51.2)	17 (41.5)	38 (92.7)
Mx	2 (4.9)	1 (2.4)	3 (7.3)

The patient characteristics and the distribution into the two groups post-stratification based on *CTNNB1* expression are shown.

*TCGA* The Cancer Genome Atlas.

**Fig. 1:**
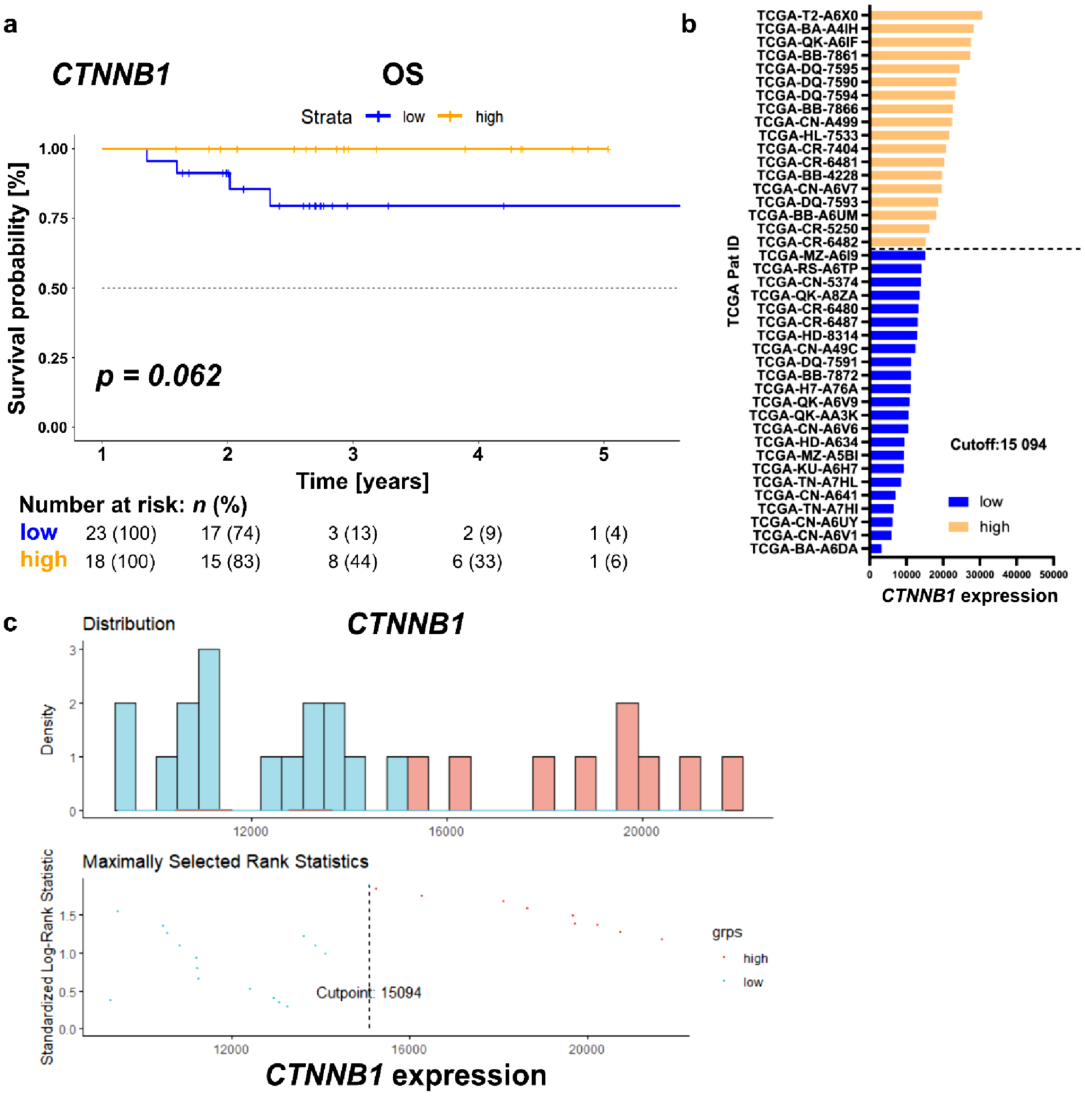
*CTNNB1* expression allows the identification of HPV-positive HNSCC patients with longer overall survival. **a** Kaplan–Meier survival curve for patients with HPV-positive HNSCC extracted from the TCGA database and stratified according to the OTV of *CTNNB1* into “low” and “high” group. **b** Respective *CTNNB1* expression values per patient stratified into the two groups (“low” [blue] and “high” [orange]) according to the OTV (indicated as “Cutoff”) for OS. **c** Top—Distribution of *CTNNB1* expression in the two patient groups Bottom—Standardized Log-Rank statistics across multiple CTNNB1 expression values, indicating that a cutoff of 15,094 yields the highest prognostic power. *HPV* human papillomavirus, *TCGA* The Cancer Genome Atlas, *OTV* optimized threshold value, *grps* groups

After stratification by the OTV of *CTNNB1*, high *CTNNB1* expression showed a trend of being associated with longer OS (Fig. 1, median OS for both groups not reached, 95% confidence interval (CI) not applicable, log-rank *p* = *0.062*). The distribution of the patients across the two groups is shown in Fig. 1b, c.

### High β-CATENIN expression predicts a better overall survival—in-house cohort

Based on the in silico analysis of *CTNNB1* expression in HPV-positive HNSCC patients of the TCGA cohort, we sought to evaluate the expression levels and prognostic value of β-CATENIN on protein level. A collection of HPV-positive HNSCC cases, primarily surgically treated at the General Hospital of Vienna, was screened. A total of 31 patients with available follow-up fulfilled the inclusion criteria. Table 2 illustrates the detailed patient characteristics of the in-house cohort. Thirteen patients were female (41.9%). The median age of the cohort was 64 years (range 37.0–80.5 years). A small subset of patients presented with advanced local disease (T3-4, *n* = *5*, 16.1%). Furthermore, none of the patients had distant metastases during the initial work-up. All patients underwent primary surgical resection, from which 23 patients (74.2%) received post-operative radiotherapy (PORT). The median OS and DFS for all patients were 1.8 years (range 0.3–12.3 years) and 1.5 years (range 0.0–9.8 years), respectively.

**Table 2:** Patient and tumor characteristics of the in-house cohort

Characteristic	Number of patients	Percentage (%)
Gender		
Female	13	41.9
Male	18	58.1
T stage		
T4	4	12.9
T3	1	3.2
T2	17	54.9
T1	9	29
N stage		
N3	1	3.2
N2	15	48.4
N1	10	32.3
N0	5	16.1
M stage		
M0	31	100
Grading		
G3	10	32.3
G2	20	64.5
G1	1	3.2
PORT		
Yes	23	74.2
No	8	25.8

*PORT* post-operative radiotherapy

**Fig. 2:**
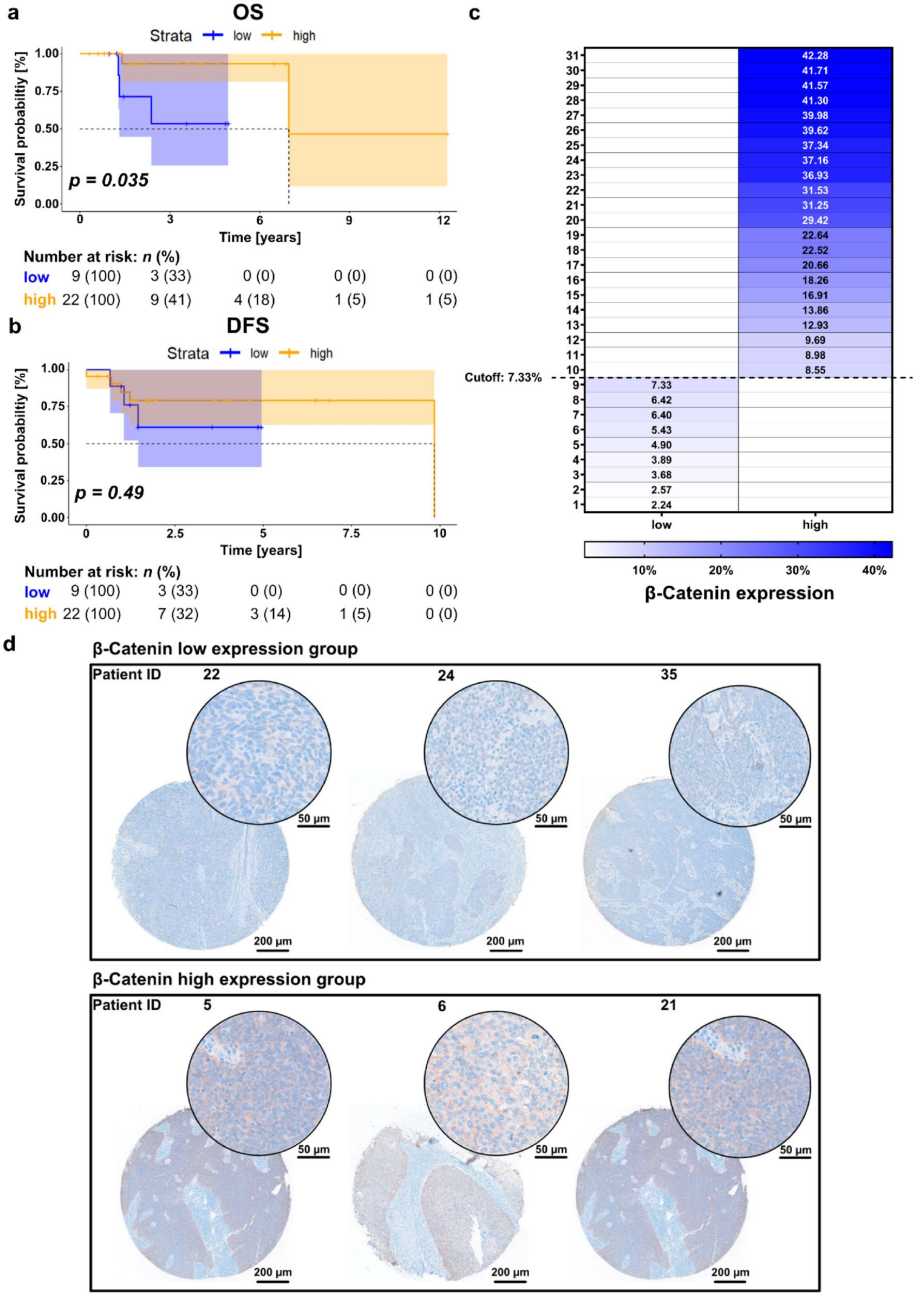
β-CATENIN is heterogeneously expressed in cancer samples of HPV-positive HNSCC and predicts favorable overall survival. **a**, **b** Kaplan–Meier survival curve for OS (**a**) and DFS (**b**) stratified into low and high expression according to the OTV of β-CATENIN expression. 95% confidence intervals are visualized by the shaded areas in the Kaplan–Meier survival curves. **c** Heatmap illustrating  the percentage of cells positive for β-CATENIN expression per patient stratified into the two groups. “Low” (left column) and “high” (right column) expression were stratified by the OTV (indicated as “Cutoff” in the figure). **d** Representative IHC images for patients with “low” (top row) and “high” (bottom row) β-CATENIN expression according to the OTV. The image of the whole TMA core was acquired at a 5 × magnification and the close-up was acquired at a 20 × magnification. *OS* overall survival, *DFS* disease-free survival, *OTV* optimized threshold value, *IHC* immunohistochemistry staining, *TMA* tissue microarray

Similar to the TCGA analysis on mRNA level, longer OS times were observed in the group with high β-CATENIN expression with the OTV as cutoff (Fig. 2a, median OS for low group not reached, median OS for high group: 6.97 years, log-rank *p* = *0.035*). However, an association between β-CATENIN expression and DFS was not observed (Fig. 2a, median OS for low group not reached, median OS for high group: 9.84 years, log-rank *p* = *0.49*). The distribution of the percentage of cells showing positive staining and their relation to the two groups (stratification based on OTV) is shown in Fig. 2c. Figure 2d shows representative images of the IHC staining for three patients per group at two different magnifications.

## Discussion

In the present study, we provided novel evidence that high *CTNNB1* expression was associated with better OS in patients with HPV-associated HNSCC. Furthermore, high β-CATENIN protein levels were associated with a favorable OS in an independent in-house cohort. Hence, β-CATENIN expression could facilitate the identification of HPV-positive HNSCC patients with a better prognosis, potentially providing an easily available tool for identification of de-escalation treatment candidates. β-CATENIN is a pivotal component and positive regulator of the WBC pathway, and its prognostic value has been demonstrated in various cancer entities (Ellison et al. 2005; Kamposioras et al. 2013; Nagy et al. 2017). However, reports of its prognostic value in HNSCC patients are rare, particularly for HPV-positive HNSCC. Therefore, we evaluated whether HPV-positive HNSCC patients can be effectively stratified based on their *CTNNB1*/β-CATENIN expression. Interestingly, our analysis revealed that high *CTNNB1*/β-CATENIN expression is associated with better OS times. Similarly, high β-CATENIN was shown to associate with a lower risk of death in pancreatic cancer (Saukkonen et al. 2016) and in surgically treated colorectal patients, Kamposioras et al*.* (Kamposioras et al. 2013) found a positive association between β-CATENIN expression and DFS. High viral load is associated with better survival in patients with HPV-associated cancers. This fact might explain why β-CATENIN, which expression is modulated by the viral oncoproteins E6 and E7, has a similar prognostic effect (Deng et al. 2015; Hashida et al. 2021). Specifically, Rampias et al*.* showed that β-CATENIN expression is positively regulated by the viral oncoproteins E6 and E7. Hence, a high viral load might lead to an E6- and E7-mediated increase in β-CATENIN expression. Despite this evidence, further studies investigating the exact mechanistic nature of these associations are needed.

As described previously, patients with HPV-positive OPSCC generally have a good prognosis. Therefore, strategies for treatment de-escalation have been investigated (Mirghani and Blanchard 2018; Bonomo and Livi 2020). These treatment de-escalation regimes should decrease treatment-associated morbidity while maintaining comparable efficacy. Since *CTNNB1*/β-CATENIN was overexpressed in patients with good prognosis, potential therapies tailored toward β-CATENIN-expressing cells, such as PRI-724 (a small molecule inhibitor of the interaction between CBP and β-CATENIN), might be a viable option for treatment de-escalation for these HPV-positive HNSCC patients (Zhang and Wang 2020).

The limited size of the TCGA and the in-house cohort are evident limitations of the study. This is attributable to the circumstance that only about 25% of all diagnosed HNSCC cases are associated with HPV (Tumban 2019; Dong et al. 2021). Nonetheless, a trend toward a significant association of high *CTNNB1* expression and a better survival outcome was observed in the TCGA cohort. Furthermore, the hypothesis that high β-CATENIN expression is associated with a better OS could be validated on protein level in our independent in-house cohort. Lastly, there are currently no standardized methods for differentiation between active and latent HPV infection in HNSCC. However, as the HPV positivity was shown by FISH, we can hypothesize that the HPV infection was active in all used tissue samples (Gkolfinopoulos et al. 2021).

Especially due to the above-mentioned weak point of the study, further investigations (including functional assessments of the role of β-CATENIN in HPV-positive HNSCC and studies exploring the prognostic value of β-CATENIN in larger patient cohorts) on the prognostic value of β-CATENIN expression are recommended. This could enable the establishment of an additional prognostic marker (in addition to the HPV status), which would facilitate timely risk stratification of HPV-associated HNSCC patients and ultimately facilitate identifying de-escalation treatment candidates.

## Supplementary Information

Below is the link to the electronic supplementary material.

Supplementary file1 (DOCX 358 KB)

## Data Availability

The public dataset supporting the conclusions of this article is available in the TCGA repository [TCGA-HNSC, https://portal.gdc.cancer.gov/projects/TCGA-HNSC].

## Abbreviations

CBP: Creb-binding protein
DFS: Disease-free survival
FFPE: Formalin-fixed paraffin-embedded
HNSCC: Head and neck squamous cell carcinoma
HPV: Human papillomavirus
IHC: Immunohistochemistry
OPSCC: Oropharyngeal squamous cell carcinoma
OS: Overall survival
OTV: Optimized threshold value
PORT: Postoperative radiotherapy
Siah-1: Seven in absentia homologue
TCGA: The Cancer Genome Atlas
TMA: Tissue microarray
WBC: Wnt/β-Catenin
