# Supplementary material for: β-CATENIN is a positive prognostic marker for HPV-positive head and neck squamous cell carcinoma
Source: J Cancer Res Clin Oncol. 2023 Apr 3;149(10):7743–50. doi: 10.1007/s00432-023-04712-3 (PMC10374714; doi:10.1007/s00432-023-04712-3)
Supplement: Supplementary file 1 — Supplementary file1 (DOCX 358 KB) [file 432_2023_4712_MOESM1_ESM.docx]

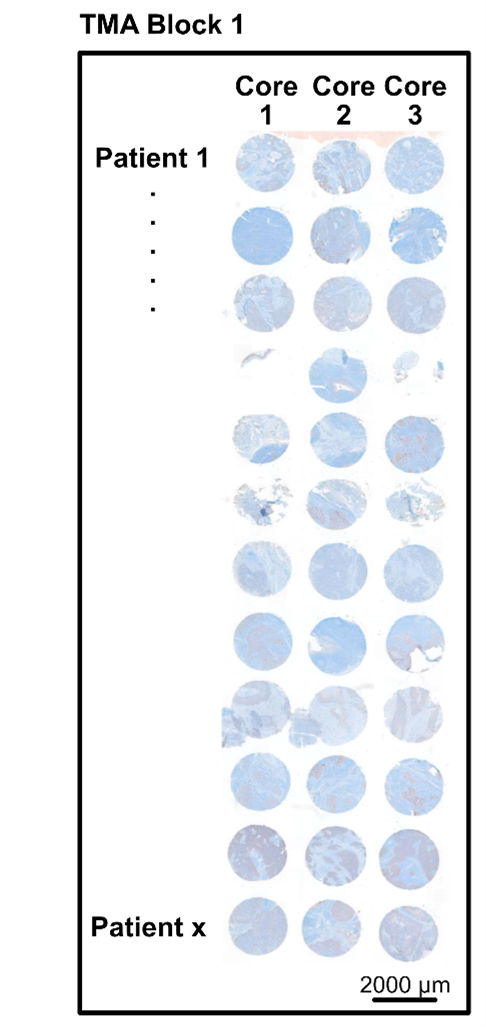


Supplementary Fig 1: Representative layout of the TMA used to validate β-CATENIN expression in HPV-positive HNSCC patients. Every row represents one patient and the three TMA cores (per row) reflect three representative tissue replicates from the primary tumor of a patient. TMA; tissue microarray
